# Design and Analysis of a Novel Fully Decoupled Tri-axis Linear Vibratory Gyroscope with Matched Modes

**DOI:** 10.3390/s150716929

**Published:** 2015-07-13

**Authors:** Dunzhu Xia, Lun Kong, Haiyu Gao

**Affiliations:** Key Laboratory of Micro Inertial Instruments and Advanced Navigation Technology of the Ministry of Education, School of Instrument Science and Engineering, Southeast University, Nanjing 210096, China; E-Mails: konglun_2015@163.com (L.K.); 101010203@seu.edu.cn (H.G.)

**Keywords:** tri-axis gyroscope, gyroscope structure, PSO algorithm, mode matching

## Abstract

We present in this paper a novel fully decoupled silicon micromachined tri-axis linear vibratory gyroscope. The proposed gyroscope structure is highly symmetrical and can be limited to an area of about 8.5 mm × 8.5 mm. It can differentially detect three axes’ angular velocities at the same time. By elaborately arranging different beams, anchors and sensing frames, the drive and sense modes are fully decoupled from each other. Moreover, the quadrature error correction and frequency tuning functions are taken into consideration in the structure design for all the sense modes. Since there exists an unwanted in-plane rotational mode, theoretical analysis is implemented to eliminate it. To accelerate the mode matching process, the particle swam optimization (PSO) algorithm is adopted and a frequency split of 149 Hz is first achieved by this method. Then, after two steps of manual adjustment of the springs’ dimensions, the frequency gap is further decreased to 3 Hz. With the help of the finite element method (FEM) software ANSYS, the natural frequencies of drive, yaw, and pitch/roll modes are found to be 14,017 Hz, 14,018 Hz and 14,020 Hz, respectively. The cross-axis effect and scale factor of each mode are also simulated. All the simulation results are in good accordance with the theoretical analysis, which means the design is effective and worthy of further investigation on the integration of tri-axis accelerometers on the same single chip to form an inertial measurement unit.

## 1. Introduction

Micromachined gyroscopes are an important kind of inertial sensor used to measure the angular rate or attitude angle. They have been developed rapidly in recent years as widely used miniaturized angular rate sensors. Since they have the merits of small volume, light weight, high reliability, low cost and potential for mass production, micromachined gyroscopes are available for various applications like aerospace measurement, balance control, inertial navigation, and the electronic stability programs, *etc.* [1,2,3]. However, most researchers are concentrating on researching single axis gyroscope at present, especially in-plane *z*-axis gyroscopes. Tri-axis gyroscopes attract less attention for their complexity, though they are the development trend of the future, and an indispensable part of an inertial measurement unit.

In a tri-axis gyroscope, the lateral-axis sensing needs an out-of-plane movement. Compared with an in-plane *z*-axis silicon micromachined gyroscope which is available for inertial-grade applications, the lateral-axis gyroscope faces more challenges in achieving good performance. Therefore, one of the difficulties in designing a tri-axis gyroscope lies in the design of lateral-axis sensing. Researchers from Seoul National University proposed a *x*-axis gyroscope with vertical drive and in-plane sensing in 2005 [4]. It was compatible with the *z*-axis gyroscope fabrication process. In 2010, Guo *et al.* from Peking University designed a lateral-axis silicon micromachined tuning fork gyroscope [5]. The proposed gyroscope has lateral drive and torsional *z*-sensing. Researchers from Peking University also proposed a vibrating wheel lateral-axis gyroscope in 2011 [6]. These innovative gyroscope structures for lateral-axis sensing can provide the inspiration for the design of a tri-axis gyroscope.

At present, the reported silicon micromachined tri-axis gyroscopes can be divided into two categories. One kind of tri-axis gyroscope is achieved by integrating three single axis gyroscopes together. In this case, the cross-axis errors can be dramatically reduced compared to a compact single proof mass or mechanically-coupled design. If the alignment error during the assembling or packaging can be well restricted within a small range, this kind of tri-axis gyroscope can achieve high precision but with high cost. Some high-performance tri-axis products available on the market are implemented by assembling three single axis gyroscopes together [7,8]. Researchers from Aalto University presented the reliability assessment of a tri-axis gyroscope under various shock loading conditions [9]. The gyroscope in their study was composed of three *z*-axis gyroscopes packaged together orthogonal to each other. Scientists from France designed a single-chip tri-axis capacitive gyroscope composed of one *z*-axis gyroscope and two *x*/*y*-axes gyroscopes in 2013 [10]. The other kind of tri-axis gyroscope is implemented on a single-chip with an ingenious structure. Vigna *et al.* from STMicroelectronics developed a single-chip tri-axis gyroscope with a compact mechanical design that combines a triple tuning-fork structure within a single vibrating element [11]. Though the cross-axis errors are not suppressed, it fulfills the pressing market requirements for small size, low power consumption and low cost. Tsai from National Cheng Kung University in Taiwan carefully designed a novel vibrating wheel tri-axis gyroscope fabricated on a single-chip [12]. The *x* and *y*-axes angular rates are detected by out-of-plane torsional plates, while the z-axis rotation detection is achieved by in-plane comb sensing. Jiao *et al.* from the Shanghai Institute of Microsystems and Information Technology proposed a delicate tri-axis gyroscope design on a single-chip [13]. The design is implemented with novel tetrapendulum proof masses for the *x*-, *y*-axes and regular proof masses for *z*-axis rate sensing. Three-axis angular rates can be sensed by the tri-axis gyroscopes in these designs, making them all valuable for future work in the design of tri-axis gyroscopes. 

The mechanical coupling between the drive and sense modes of a gyroscope has a great impact on its performance, including the bias stability and dynamic range, *etc.* [14]. The decoupling structure is widely adopted by researchers in *z*-axis gyroscopes. Performance of the gyroscope can be dramatically improved when compared to that possible without utilizing any decoupling mechanism [15]. Shkel *et al.* from UC Irvine proposed a silicon MEMS quadruple mass gyroscope with perfectly symmetric decoupling structure, resulting in matched modes, high quality factor and good temperature characterization [16,17]. Though it is a single axis gyroscope, the concept of a symmetric decoupling structure can inspire the design of multi-axis gyroscopes. Thus in a tri-axis gyroscope, it is necessary to decouple the drive and sense modes to achieve a relatively good performance. Moreover, mode matching is essential to further improve the performance of a tri-axis gyroscope [18]. Besides, the quadrature error which is introduced by fabrication imperfection will significantly influence the performance of a gyroscope. All these factors should be taken into consideration in the design of a high-performance tri-axis gyroscope. Therefore, based on the previous studies, a novel fully decoupled tri-axis linear vibratory gyroscope on a single-chip with matched modes is proposed and analyzed in this paper. The rest of the paper is organized as four sections. The process of the structure design and principle analysis are described in Section 2. The mode matching process of the tri-axis gyroscope is discussed in Section 3. Section 4 summarizes the simulation results of the gyroscope. Section 5 presents the conclusions.

## 2. Structure Design and Analysis

### 2.1. Design of the Fully Decoupled Gyroscope

**Figure 1:**
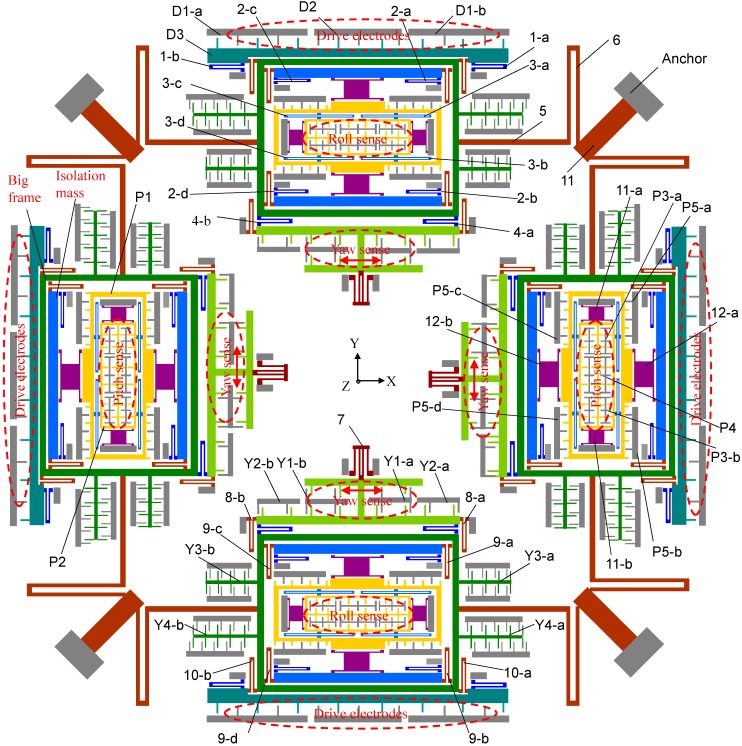
Schematic of the proposed vibratory tri-axis gyroscope. (1) Drive mode springs: U-shaped spring-1, spring-2, spring-4, double-U-shaped spring-3, crab-leg spring-5; D1: driving electrodes; D2: drive-sense electrode; D3: drive beam. (2) Yaw mode springs: U-shaped spring-6, spring-8, spring-9, spring-10 and double folded spring-7; Y1: yaw-sense electrodes; Y2: feedback electrode in yaw mode; Y3: frequency tuning electrodes in yaw mode; Y4: quadrature error correction electrodes in yaw mode; (3) Pitch/roll mode springs: out-of-plane decoupling spring-11 and spring-12; P1: outer frame in roll mode; P2: inner frame in pitch mode; P3: pitch-sense electrodes; P4: feedback electrode in pitch mode; P5: quadrature error correction electrodes in pitch mode.

The schematic of the proposed tri-axis gyroscope structure is shown in Figure 1 and summarized in Table 1. It is a highly symmetric structure consisting of four identical big frames distributed on the four sides of the structure. Different kinds of springs are adopted in the structure to achieve the goal of full decoupling. The working principle of the gyroscope can be described as follows: 

(1) *The drive mode*: the gyroscope is designed to have commonly comb driving with the drive beam arranged at the periphery of the big frame. When a driving voltage is applied on the comb drive electrodes D1, all the driving parts including the big frame, the isolation mass for yaw-pitch/roll modes, and the outer frame P1 in pitch/roll mode are driven to move together with the drive beam in the drive direction. The drive mode movement looks like a “beating heart”, which means the drive mass in the four sides will move towards or away from the center of the structure at the same time. Moreover, both the inner frame R2 (sense frame) in pitch/roll mode and the yaw beam will not be driven to move by the driving force as the corresponding springs (including spring-11, -7 and -8) connected with anchors have very big stiffness in the drive direction. 

(2) *The yaw mode*: when an angular rate is exerted on the gyroscope about *z*-axis, the big frame will generate an in-plane translational movement orthogonal with the drive direction due to the Coriolis effect. Then the yaw beam will be driven to move together with the big frame in the yaw sense direction. There are four yaw beams distributed at the outside of each big frame. The *z*-axis angular rate is designed to be differentially detected by parallel plate electrodes Y1-a and Y1-b. Y2-a and Y2-b are the feedback electrodes in yaw mode. 

**Table 1:** Summary of part numbers and function description in three modes.

Part Number	Function Description	
1-a, 1-b, 1-c, 1-d	U-shaped spring	In drive mode
2-a, 2-b, 2-c, 2-d		
3-a, 3-b, 3-c, 3-d	Double-U-shaped spring	
4-a, 4-b	U-shaped spring	
5	Crab-leg spring	
D1-a, D1-b	Driving electrodes	
D2	Drive-sense electrode	
6	U-shaped spring	In yaw mode
7	Double folded spring	
8-a, 8-b	U-shaped spring	
9-a, 9-b, 9-c, 9-d		
10-a, 10-b		
Y1-a, Y1-b	Sense electrodes	
Y2-a, Y2-b	Feedback electrode	
Y3-a, Y3-b	Frequency tuning electrodes	
Y4-a, Y4-b	Quadrature error correction electrodes	
11-a, 11-b	Out-of-plane decoupling spring	In pitch/roll mode
12-a, 12-b		
P1	Outer frame	
P2	Inner frame	
P3-a, P3-b	Sense electrodes	
P4	Feedback electrode	
P5-a, P5-b, P5-c, P5-d	Quadrature error correction electrodes	

Besides, the frequency tuning electrodes Y3 and the quadrature error correction electrodes Y4 are arranged at the two sides of the big frame respectively. Obviously, the yaw beam has only 1-DOF (degree of freedom) in the sense direction, thus it is a mechanical decoupling structure.

**Figure 2:**
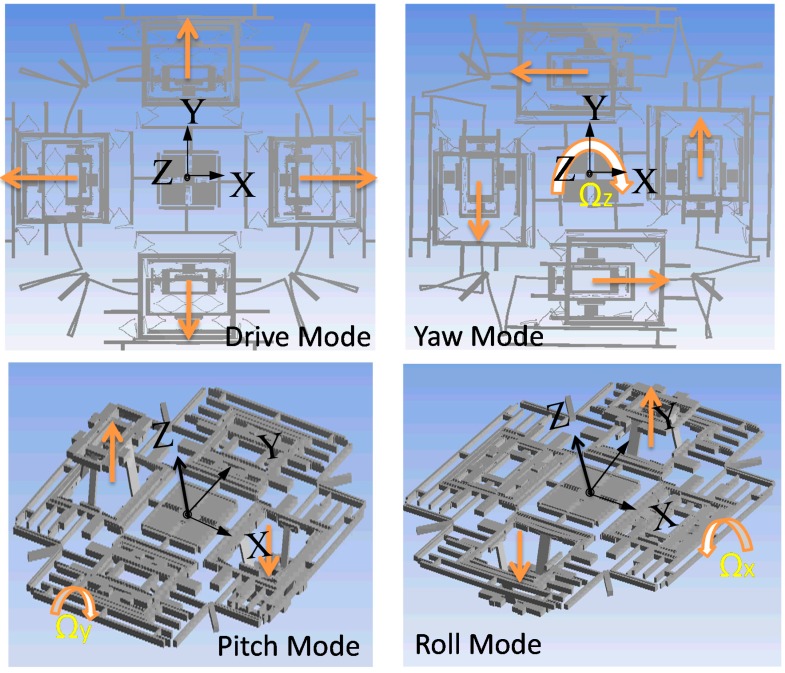
Schematic diagrams for drive mode, yaw mode and pitch/roll mode.

(3) *The pitch mode*: the pitch mode angular velocity is differentially detected by the two big frame structures in *y*-axis shown in Figure 1. The outer frame P1 in pitch/roll mode has 2-DOF in the in-plane drive direction and out-of-plane *z*-sensing direction. When there is a *x*-axis angular velocity applied on the gyroscope, the inner frame in pitch/roll mode P2 will be driven to move together with the outer frame P1 in *z*-axis direction through the double U-shaped spring-3. The sensing electrodes P3-a and P3-b in pitch/roll mode are designed to be comb fingers which have different thickness in *z*-axis, so that the capacitance change is proportional to the *z*-axis movement caused by Coriolis force. P4 is the feedback electrode placed in the inner frame P2. P5 are the quadrature error correction electrodes arranged at the outer frame P1. Besides, the frequency tuning electrode in pitch/roll mode can be a plate placed under the outer frame P1.

(4) *The roll mode*: the rotation velocity in the roll mode is differentially detected by the two big frame structures in *x*-axis. Its working principle is same with the pitch mode. In this design, the fully decoupled mechanism depends on the elaborately arranged springs. As a result, the drive beam, yaw beam and inner frame in pitch/roll mode have only 1-DOF in their own drive or sense direction, respectively. The key point in the structure design lies in the decoupling of the in-plane movement in drive direction and the out-of-plane movement in pitch/roll mode. Thus the out-of-plane-decoupling spring-11 and -12 with thinner thickness than the other structures are adopted. They have relatively small stiffness in the *z*-axis direction and very big stiffness in the lateral axis direction, and thus can be used in the pitch/roll mode to achieve drive-to-sense decoupling. The schematic diagrams for different modes of motion are shown in Figure 2.

### 2.2. Quadrature Error Correction

Quadrature error introduced by fabrication imperfection is actually the stiffness coupling from the drive mode to the sense mode [19]. When the input angular rate is small, the quadrature error signal can be hundreds of times greater than the associated Coriolis signal. To eliminate the quadrature error as much as possible, a closed-loop sense circuit or a structural correction method is needed. However, it is difficult to implement an exact circuitry control system including the frequency control, phase control and amplitude control in a tri-axis gyroscope closed-loop sense circuit. Therefore, it is necessary to design a tri-axis gyroscope structure with quadrature error correction function in all the sense modes.

#### 2.2.1. Quadrature Error Correction in Yaw Mode

**Figure 3:**
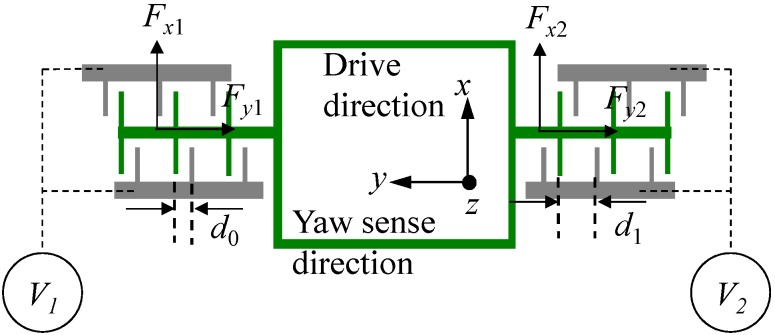
The quadrature error correction electrodes in yaw mode.

The quadrature error correction electrodes of yaw mode are arranged at the outside of the big frame. The working principle of the quadrature error correction is described in Figure 3. Assuming that two direct voltages *V*_1_ and *V*_2_ are exerted on the fixed electrodes on two sides of the big frame, the electrostatic energy of the parallel plates in one side can be expressed as:

(1) $$E_{1}=\frac{1}{2}\frac{n\varepsilon h(l-x)}{d_{0}-y}V_{1}^{2}+\frac{1}{2}\frac{n\varepsilon h(l-x)}{d_{1}+y}V_{1}^{2}+\frac{1}{2}\frac{n\varepsilon h(l+x)}{d_{0}+y}V_{1}^{2}+\frac{1}{2}\frac{n\varepsilon h(l+x)}{d_{1}-y}V_{1}^{2}$$

where *ε* is the dielectric constant, *n* denotes the number of parallel plates; *h* is the thickness of the structure; *l* is the overlap length of the parallel plates; *d*_0_ and *d*_1_ denote the gaps of the parallel plates; *x* and *y* are the displacement in *x* and *y*-axes respectively. 

The electrostatic force in *x* and *y*-axes can be calculated from Equation (1) as:

(2) $$\left\{ \begin{array}{l} F_{x1}=\frac{\partial E_{1}}{\partial x}=0\\ F_{y1}=\frac{1}{2}\frac{\partial E_{1}}{\partial y}=\frac{1}{2}n\varepsilon h\frac{4ld_{0}y-2(d_{0}^{2}+y^{2})x}{{\left(d_{0}+y\right)}^{2}{\left(d_{0}-y\right)}^{2}}V_{1}^{2}+\frac{1}{2}n\varepsilon h\frac{4ld_{1}y+2(d_{1}^{2}+y^{2})x}{{\left(d_{1}+y\right)}^{2}{\left(d_{1}-y\right)}^{2}}V_{1}^{2} \end{array}\right.$$

Considering that the displacement *y* in the sense mode is orders of magnitude smaller than the gap of the parallel plates, it can be ignored to simplify the analysis. Thus the electrostatic force in *y*-axis can be simplified as:

(3) $$F_{y1}=n\varepsilon h(\frac{1}{d_{1}^{2}}-\frac{1}{d_{0}^{2}})V_{1}^{2}x$$

Similarly, the electrostatic force of quadrature error correction electrodes in another side of the big frame can be solved:

(4) $$F_{x2}=0,F_{y2}=n\varepsilon h(\frac{1}{d_{0}^{2}}-\frac{1}{d_{1}^{2}})V_{2}^{2}x$$

Thus the electrostatic force generated by quadrature error correction electrodes in both sides of the big frame can be expressed as:

(5) $$\left\{ \begin{array}{l} F_{x}=F_{x1}+F_{x2}=0\\ F_{y}=F_{y1}+F_{y2}=n\varepsilon h(\frac{1}{d_{0}^{2}}-\frac{1}{d_{1}^{2}})(V_{2}^{2}-V_{1}^{2})x \end{array}\right.$$

Obviously, the quadrature force in *y*-axis induced by the stiffness coupling from drive mode can be cancelled out by the electrostatic force according to Equation (5). By changing the values of the direct voltages *V*_1_ and *V*_2_ exerted on the correction electrodes, the quadrature error can be offset flexibly. While the electrostatic force in *x*-axis is zero, it does not have any impact on the performance of the gyroscope.

#### 2.2.2. Quadrature Error Correction in Pitch/Roll Mode

**Figure 4:**
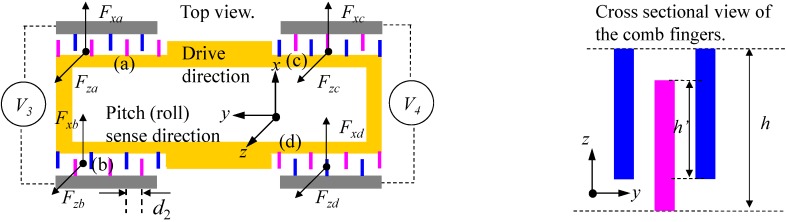
The quadrature error correction electrodes in pitch/roll mode.

The quadrature error correction electrodes of pitch/roll mode lie in the pitch-outer-frame as shown in Figure 1. The working principle of the quadrature error correction can be described in Figure 4. Assuming two direct voltages *V*_3_ and *V*_4_ are exerted on the fixed electrodes on two sides of the frame, the electrostatic energy of the comb fingers can be expressed as:

(6) $$\left\{ \begin{array}{l} E_{a}=\frac{n^{\prime }\varepsilon (h^{\prime }+z)(l^{\prime }+x)}{d_{2}}V_{3}^{2},E_{b}=\frac{n^{\prime }\varepsilon (h^{\prime }-z)(l^{\prime }-x)}{d_{2}}V_{3}^{2}\\ E_{c}=\frac{n^{\prime }\varepsilon (h^{\prime }-z)(l^{\prime }+x)}{d_{2}}V_{4}^{2},E_{d}=\frac{n^{\prime }\varepsilon (h^{\prime }+z)(l^{\prime }-x)}{d_{2}}V_{4}^{2} \end{array}\right.$$

where *n'* is the number of comb fingers; *h'* is the overlap thickness of the comb fingers; *l'* is the overlap length of the comb fingers; *d*_2_ is the comb finger gap; *x* and *z* are the displacements in *x* and *z*-axes respectively.

The electrostatic force in *x* and *z*-axes can be calculated from Equation (6) as:

(7) $$\left\{ \begin{array}{l} F_{xa}=\frac{n^{\prime }\varepsilon (h^{\prime }+z)}{d_{2}}V_{3}^{2},F_{za}=\frac{n^{\prime }\varepsilon (l^{\prime }+x)}{d_{2}}V_{3}^{2}\\ F_{xb}=-\frac{n^{\prime }\varepsilon (h^{\prime }-z)}{d_{2}}V_{3}^{2},F_{zb}=-\frac{n^{\prime }\varepsilon (l^{\prime }-x)}{d_{2}}V_{3}^{2}\\ F_{xc}=\frac{n^{\prime }\varepsilon (h^{\prime }-z)}{d_{2}}V_{4}^{2},F_{zc}=-\frac{n^{\prime }\varepsilon (l^{\prime }+x)}{d_{2}}V_{4}^{2}\\ F_{xd}=-\frac{n^{\prime }\varepsilon (h^{\prime }+z)}{d_{2}}V_{4}^{2},F_{zd}=\frac{n^{\prime }\varepsilon (l^{\prime }-x)}{d_{2}}V_{4}^{2} \end{array}\right.$$

Then the resultant force generated by quadrature error correction electrodes can be expressed as:

(8) $$\left\{ \begin{array}{l} {F_{x}}^{\prime }=F_{xa}+F_{xb}+F_{xc}+F_{xd}=\frac{2n^{\prime }\varepsilon }{d_{2}}(V_{3}^{2}-V_{4}^{2})z\approx 0\\ F_{z}=F_{za}+F_{zb}+F_{zc}+F_{zd}=\frac{2n^{\prime }\varepsilon }{d_{2}}(V_{3}^{2}-V_{4}^{2})x \end{array}\right.$$

According to Equation (8), the electrostatic force *F_x_'* in *x*-axis generated by the quadrature error correction electrodes can be ignored as the displacement in *z*-axis is orders of magnitude smaller than that in *x*-axis. At the same time, the quadrature error in pitch/roll mode can be cancelled out by changing the values of the direct voltages *V*_3_ and *V*_4_.

### 2.3. Frequency Tuning

To improve the performance of a tri-axis gyroscope, the natural frequencies in the drive and sense modes should be matched. However, even though the resonant frequencies can be matched in simulation results, a frequency split still exists due to the fabrication imperfections. To offset the frequency drifts induced by fabrication errors, stiffness tuning methods should be adopted in the structure design. 

#### 2.3.1. Frequency Tuning in Yaw Mode

**Figure 5:**
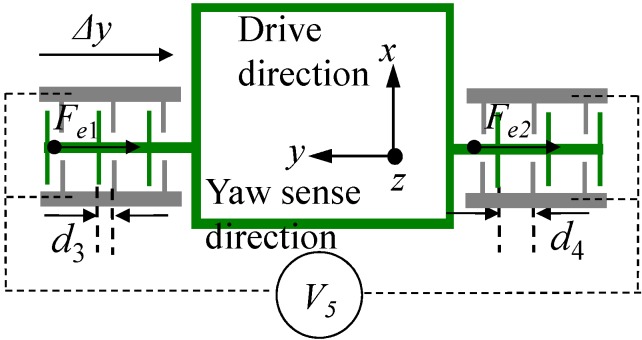
The frequency tuning electrodes in yaw mode.

The parallel plate electrodes for frequency tuning in yaw mode are arranged at the two sides of the big frame as depicted in Figure 1. The principle of frequency tuning can be described in Figure 5. Assuming that a direct voltage *V*_5_ is applied on the frequency tuning electrodes, the electrostatic force can be expressed as:

(9) $$\left\{ \begin{array}{l} F_{e1}=\frac{N\varepsilon S}{2}\left[\frac{1}{{\left(d_{3}-\Delta y\right)}^{2}}-\frac{1}{{\left(d_{4}+\Delta y\right)}^{2}}\right]V_{5}^{2}\\ F_{e2}=\frac{N\varepsilon S}{2}\left[\frac{1}{{\left(d_{4}-\Delta y\right)}^{2}}-\frac{1}{{\left(d_{3}+\Delta y\right)}^{2}}\right]V_{5}^{2} \end{array}\right.$$

where *N* is the number of parallel plates; *S* is the overlap area of a pair of parallel plates; *d*_3_ and *d*_4_ denote the gaps of the parallel plates; Δ*y* is the displacement in *y*-axis.

Then the resultant force generated by the frequency tuning electrodes is:

(10) $$\begin{array}{c} F_{e}=F_{e1}+F_{e2}=\frac{N\varepsilon SV_{5}^{2}}{2}\left[\frac{4d_{3}\Delta y}{{\left(d_{3}-\Delta y\right)}^{2}{\left(d_{3}+\Delta y\right)}^{2}}+\frac{4d_{4}\Delta y}{{\left(d_{4}-\Delta y\right)}^{2}{\left(d_{4}+\Delta y\right)}^{2}}\right]\\ \approx 2N\varepsilon SV_{5}^{2}(\frac{1}{{d_{3}}^{3}}+\frac{1}{{d_{4}}^{3}})\Delta y \end{array}$$

Because the electrostatic force *F_e_* is in the reverse direction of the elastic force, the electrostatic stiffness can be expressed as:

(11) $$k_{e}=-\frac{F_{e}}{\Delta y}=-2N\varepsilon SV_{5}^{2}(\frac{1}{{d_{3}}^{3}}+\frac{1}{{d_{4}}^{3}})$$

#### 2.3.2. Frequency Tuning in Pitch/Roll Mode

Since the pitch/roll sense frame has an out-of-plane movement, the frequency tuning electrode should be a plate located under the pitch sense frame. Assuming that a direct voltage *V*_6_ is applied on the frequency tuning electrodes, the electrostatic force can be expressed as:

(12) $$F_{ez}=\frac{1}{2}\frac{\varepsilon S^{\prime }}{{\left(d_{5}-\Delta z\right)}^{2}}V_{6}^{2}\approx \frac{1}{2}\frac{\varepsilon S^{\prime }}{{d_{5}}^{3}}(d_{5}+2\Delta z)V_{6}^{2}=\underset{const}{\underset{︸}{\frac{1}{2}\frac{\varepsilon S^{\prime }}{{d_{5}}^{2}}V_{6}^{2}}}+\frac{\varepsilon S^{\prime }}{{d_{5}}^{3}}V_{6}^{2}\Delta z$$

where *S’* is the overlap area of the plate electrode and the pitch sense frame; *d*_5_ is the gap between the plate and the pitch sense frame in *z*-axis; Δ*z* is the displacement of the pitch sense frame in *z*-axis.

Obviously, the electrostatic force *F_ez_* has a reverse direction compared with the elastic force. Moreover, there is a constant part in the expression of *F_ez_*, which will not affect the frequency in pitch mode. Thus the electrostatic stiffness can be expressed as:

(13) $$k_{ez}=-\frac{\varepsilon S^{\prime }}{{d_{5}}^{3}}V_{6}^{2}$$

### 2.4. Elimination of Rotational Movement

Based on the proposed schematic structure in Figure 1, the dimensions of the designed structure apart from the springs can be obtained by carefully calculating the drive force, sensitivities and masses in different modes. In this way, the purpose of designing a fully decoupled tri-axis gyroscope with reasonable driving force and uniform sensitivity in three sense modes is achieved. Part of the structure dimensions are listed in Table 2. In this case, considering the precision has a proportional relationship with the quantity of proof mass, we choose the total die size as 8500 μm by 8500 μm. This might seem relatively larger than other reported tri-axis gyroscopes, but our design goal is to realize a high precision industrial use tri-gyroscope with zero bias stability of sub 10 °/s rather than a consumer device. In our previous design, the high precision single axis gyroscope area is already about 5000 μm × 5000 μm. Further, compared with a combination of three single axis gyroscopes packaged together orthogonal to each other, our integrated tri-axis gyroscope can still have obvious volume advantages over them. Besides, the cost and alignment error of three single axis gyroscopes are both effectively reduced during the assembly or packaging.

**Table 2:** Part of the structure dimensions.

Parameter	Value
Total die size	8500 μm × 8500 μm
Structure thickness (*h*)	60 μm
Drive mode (a quarter of the structure)	
Drive beam length and width	4000 μm × 70 μm
Comb finger length, overlap length, width and gap	40 μm, 20 μm, 5 μm, 3 μm
Drive capacitance	2.69 pF
Drive-sense capacitance	0.67 pF
Big frame width	120 μm
Mass in drive mode (*m_d_*)	0.460 mg
Yaw mode (a quarter of the structure)	
Yaw beam length and width	2320 μm × 70 μm
Parallel plate length, width, and two plate gaps	120 μm, 5 μm, 3 μm and 15 μm
Yaw sense capacitance	0.74 pF
Feedback capacitance	0.11 pF
Mass in yaw mode (*m_y_*)	0.273 mg
Pitch/roll mode (a quarter of the structure)	
Comb finger length, overlap length, width and gap	100 μm, 85 μm, 5 μm, 3 μm
Comb finger thickness and overlap thickness	45 μm, 30 μm
Pitch/roll sense capacitance	0.70 pF
Feedback capacitance	0.32 pF
Mass in pitch/roll mode (*m_p_*)	0.144 mg

**Figure 6:**
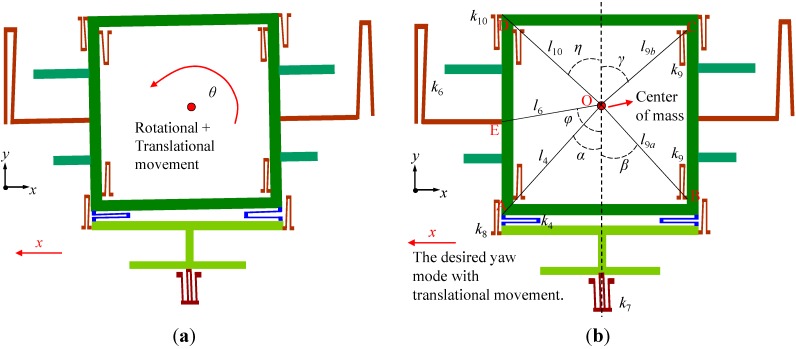
Analysis of the movement in yaw mode. (**a**) The resultant motion including the undesired rotational movement of the big frame. (**b**) The desired translational movement.

After defining the sizes of the frames in different modes, the dimensions of the springs numbered from 1 to 12 shown in Figure 1 should be further discussed and chosen for mode matching. At the beginning, the springs are chosen with their typical values and the structure is analyzed in ANSYS. It is found that the drive and pitch/roll modes can work well with the desired in-plane and out-of-plane translational movements, respectively. However, the yaw mode appears to be a resultant motion of the desired in-plane translational movement and undesired in-plane rotational movement around its center of mass as shown in Figure 6a. To avoid this undesired rotational motion, the dimensions of the springs which affect the yaw mode should be elaborately designed. The desired translational movement of yaw mode is shown in Figure 6b. Taking the rotational movement into consideration, the whole system in yaw mode can be simplified to be a 2-DOF motion system. In this case, the Lagrangian mechanics can be employed to analyze the system [20]. The generalized coordinates can be chosen to be *x* and *θ*, where *x* is the translational displacement of the big frame; *θ* is the rotational angle of the big frame around its center of mass. The Lagrange function of the 2-DOF system can be expressed as:

(14) $$L=T_{1}+T_{2}+T_{y}-U_{7}-2U_{8}-2U_{4}-2U_{9a}-2U_{9b}-2U_{6}-2U_{10}$$

where *T*_1_ and *T*_2_ are the kinetic energies of big frame under translational movement and rotational movement respectively; *T_y_* is the kinetic energy of yaw beam under translational movement; *U*_7_, *U*_8_, *U*_4_, *U*_9*a*_, *U*_9*b*_, *U*_6_, *U*_10_ are the elastic potential energies of the springs respectively; the coefficients of the elastic potential energies denote the number of different springs respectively.

The translational kinetic energy of the big frame can be expressed as:

(15) $$T_{1}=\frac{1}{2}m_{1}{\dot{x}}^{2}$$

where *m*_1_ is the mass of the big frame.

The rotational kinetic energy of the big frame can be expressed as:

(16) $$T_{2}=\frac{1}{2}I_{0}{\dot{\theta }}^{2}$$

where *I*_0_ is the moment of inertia of the big frame around its center of mass.

Taking the rotational movement of the big frame into consideration, the translational displacement of the yaw beam can be expressed as:

(17) $$x_{y}=x-\left[l_{4}\sin\alpha -l_{4}\sin(\alpha -\theta )\right]$$

where *l*_4_ is the distance between the center of mass and the corner of spring-4; *α* is the angle between line *l*_4_ and the vertical dotted line across the center of mass as shown in Figure 6b.

The translational kinetic energy of the yaw beam can be expressed as:

(18) $$T_{y}=\frac{1}{2}m_{y}{{\dot{x}}_{y}}^{2}$$

where *m_y_* is the mass of the yaw beam.

The elastic potential energy of the springs can be expressed as:

(19) $$U_{7}=\frac{1}{2}k_{7}{x_{y}}^{2}$$

(20) $$U_{8}=\frac{1}{2}k_{8}{x_{y}}^{2}$$

(21) $$U_{4}=\frac{1}{2}k_{4}{\left[l_{4}\cos\alpha -l_{4}\cos(\alpha +\theta )\right]}^{2}$$

(22) $$U_{9a}=\frac{1}{2}k_{9}{\left[x+l_{9a}\sin\beta -l_{9a}\sin(\beta +\theta )\right]}^{2}$$

(23) $$U_{9b}=\frac{1}{2}k_{9}{\left[x+l_{9b}\sin\gamma -l_{9b}\sin(\gamma -\theta )\right]}^{2}$$

(24) $$U_{6}=\frac{1}{2}k_{6}{\left[x-l_{6}\sin\varphi +l_{6}\sin(\varphi -\theta )\right]}^{2}$$

(25) $$U_{10}=\frac{1}{2}k_{10}{\left[x-l_{10}\sin\eta +l_{10}\sin(\eta +\theta )\right]}^{2}$$

where *k*_4_ is the stiffness of spring-4 in *y*-axis; *k*_6_, *k*_7_, *k*_8_, *k*_9_ and *k*_10_ are the stiffnesses of relevant springs in *x*-axis respectively; *l*_6_, *l*_9*a*_, *l*_9*b*_ and *l*_10_ are the distances between the center of mass and the corners of springs respectively; *β*, *γ*, *φ* and *η* are the angles between lines *l*_9*a*_, *l*_9*b*_, *l*_6_, *l*_10_ and the vertical dotted line across the center of mass respectively; these parameters are depicted in Figure 6b.

Without any influence on the accuracy of analysis, the whole system can be treated as a conservative system. Thus the dynamic equations can be expressed as:

(26) $$\left\{ \begin{array}{l} \frac{\partial }{\partial t}\frac{\partial L}{\partial \dot{x}}-\frac{\partial L}{\partial x}=0\\ \frac{\partial }{\partial t}\frac{\partial L}{\partial \dot{\theta }}-\frac{\partial L}{\partial \theta }=0 \end{array}\right.$$

Substituting all the kinetic energies and the elastic potential energies into Equation (26), the Lagrange function can be expressed as a function of the generalized coordinates *x* and *θ*. Thus the dynamic equations can be calculated as:

(27) $$\frac{\partial }{\partial t}\frac{\partial L}{\partial \dot{x}}=(m_{1}+m_{y})\ddot{x}$$

(28) $$\begin{array}{l} \frac{\partial L}{\partial x}=-k_{7}\left[x-l_{4}\sin\alpha +l_{4}\sin(\alpha -\theta )\right]-2k_{8}\left[x-l_{4}\sin\alpha +l_{4}\sin(\alpha -\theta )\right]\\ -2k_{9}\left[x+l_{9a}\sin\beta -l_{9a}\sin(\beta +\theta )\right]-2k_{9}\left[x+l_{9b}\sin\gamma -l_{9b}\sin(\gamma -\theta )\right]\\ -2k_{6}\left[x-l_{6}\sin\varphi +l_{6}\sin(\varphi -\theta )\right]-2k_{10}\left[x+l_{10}\sin(\eta +\theta )-l_{10}\sin\eta \right]\\ \frac{\partial L}{\partial x}|{\;}_{\sin\theta =\theta ,\cos\theta =1}=(-2k_{6}-k_{7}-2k_{8}-2k_{9}-2k_{10})x+\\ \left[(k_{7}+2k_{8})l_{4}\cos\alpha +2k_{9}(l_{9a}\cos\beta -l_{9b}\cos\gamma )-2k_{10}l_{10}\cos\eta +2k_{6}l_{6}\cos\varphi \right]\theta \end{array}$$

(29) $$\frac{\partial }{\partial t}\frac{\partial L}{\partial \dot{\theta }}=I_{0}\ddot{\theta }$$

(30) $$\begin{array}{l} \frac{\partial L}{\partial \theta }=2k_{4}\left[l_{4}\cos\alpha -l_{4}\cos(\alpha +\theta )\right]l_{4}\sin(\alpha +\theta )+(k_{7}+2k_{8})\left[x-l_{4}\sin\alpha +l_{4}\sin(\alpha -\theta )\right]l_{4}\cos(\alpha -\theta )\\ +2k_{9}\left[x+l_{9a}\sin\beta -l_{9a}\sin(\beta +\theta )\right]l_{9a}\cos(\beta +\theta )-2k_{9}\left[x+l_{9b}\sin\gamma -l_{9b}\sin(\gamma -\theta )\right]l_{9b}\cos(\gamma -\theta )\\ -2k_{10}\left[x-l_{10}\sin\eta +l_{10}\sin(\eta +\theta )\right]l_{10}\cos(\eta +\theta )+2k_{6}\left[x-l_{6}\sin\varphi +l_{6}\sin(\varphi -\theta )\right]l_{6}\cos(\varphi -\theta )\\ \frac{\partial L}{\partial \theta }|{\;}_{\sin\theta =\theta ,\cos\theta =1}=\left[(k_{7}+2k_{8})l_{4}\cos\alpha +2k_{9}(l_{9a}\cos\beta -l_{9b}\cos\gamma )-2k_{10}l_{10}\cos\eta +2k_{6}l_{6}\cos\varphi \right]x+\\ \left[2k_{4}l_{4}^{2}\sin^{2}\alpha -(k_{7}+2k_{8})l_{4}^{2}\cos^{2}\alpha -2k_{9}(l_{9a}^{2}\cos^{2}\beta +l_{9b}^{2}\cos^{2}\gamma )-2k_{10}l_{10}^{2}\cos^{2}\eta -2k_{6}l_{6}^{2}\cos^{2}\varphi \right]\theta \end{array}$$

Then the dynamic equations can be expressed as:

(31) $$\left[\begin{array}{l} \ddot{x}\\ \ddot{\theta } \end{array}\right]=\left[\begin{array}{cc} A_{1} & A_{2}\\ A_{3} & A_{4} \end{array}\right]\left[\begin{array}{c} x\\ \theta \end{array}\right]$$

where the coefficients are:

$A_{1}=(-2k_{6}-k_{7}-2k_{8}-2k_{9}-2k_{10})/(m_{1}+m_{y})$
$A_{2}=\left[(k_{7}+2k_{8})l_{4}\cos\alpha +2k_{9}(l_{9a}\cos\beta -l_{9b}\cos\gamma )-2k_{10}l_{10}\cos\eta +2k_{6}l_{6}\cos\varphi \right]/(m_{1}+m_{y})$
$A_{3}=\left[(k_{7}+2k_{8})l_{4}\cos\alpha +2k_{9}(l_{9a}\cos\beta -l_{9b}\cos\gamma )-2k_{10}l_{10}\cos\eta +2k_{6}l_{6}\cos\varphi \right]/I_{0}$
$A_{4}=[2k_{4}l_{4}^{2}\sin^{2}\alpha -(k_{7}+2k_{8})l_{4}^{2}\cos^{2}\alpha -2k_{9}(l_{9a}^{2}\cos^{2}\beta +l_{9b}^{2}\cos^{2}\gamma )-2k_{10}l_{10}^{2}\cos^{2}\eta -2k_{6}l_{6}^{2}\cos^{2}\varphi ]/I_{0}$

In order to eliminate the rotational movement of the big frame, *i.e.*, θ = 0, the coefficients should meet the conditions of *A_2_* = 0 and *A_3_* = 0. Thus we get:

(32) $$(k_{7}+2k_{8})l_{4}\cos\alpha +2k_{9}(l_{9a}\cos\beta -l_{9b}\cos\gamma )-2k_{10}l_{10}\cos\eta +2k_{6}l_{6}\cos\varphi =0$$

Obviously, Equation (32) can be rewritten as:

(33) $$(k_{7}+2k_{8})(y_{O}-y_{A})+2k_{9}(2y_{O}-y_{B}-y_{C})-2k_{10}(y_{D}-y_{O})+2k_{6}(y_{O}-y_{E})=0$$

where *y_O_*, *y_A_* = 1468 μm, *y_B_* = 1628 μm, *y_C_* = 3520 μm, *y_D_* = 1660 μm, *y_E_* are the *y*-coordinate values of points O, A, B, C, D and E marked in Figure 6b, respectively.

## 3. Mode Matching

### 3.1. PSO Algorithm for Mode Matching

It is known that matching the drive and sense modes of a gyroscope can dramatically improve the sense mode quality factor, resulting in an enhanced sensitivity, an increased signal-to-noise ratio and a readout circuit with reduced complexity [21]. To improve the performance of a tri-axis gyroscope, it is essential to minimize the frequency split between the drive mode and three sense modes. 

As we know, the traditional dual modes matching process of a single-axis gyroscope can be implemented by iteratively adjusting manually the relevant spring dimensions and simulating the matched modes via FEM software. However, there are too many springs in this fully decoupled tri-axis gyroscope. It is complex and time consuming to realize the frequency matching process for four modes. Besides, Equation (33) should also be taken into consideration to eliminate the rotational movement of the big frame in yaw mode. Thus, we have to find an applicable algorithm to solve this problem. The PSO algorithm was developed by Eberhart and Kennedy based on the natural swarm behavior of birds and fish [22]. It is a parallel evolutionary computation technique which is available for various applications such as function optimization, system identification, fuzzy control and so on [23]. The algorithm is initialized with a set of random candidate solutions conceptualized as particles. Each particle is iteratively moved towards the location with best fitness of the particle itself and the location with best fitness of all the particles in the problem space. The algorithm will be completed when the fitness value meets the accuracy requirement within a certain number of iterations. Considering the above merits, in this case, the PSO algorithm is adopted to optimize the spring dimensions. 

To design a mode matched tri-axis gyroscope using the PSO algorithm, the objective function can be a function of the resonant frequencies in all the drive and sense modes as:

(34) $$F={\left(f_{d}-f_{obj}\right)}^{2}+{\left(f_{y}-f_{obj}\right)}^{2}+{\left(f_{p}-f_{obj}\right)}^{2}$$

where *f_d_*, *f_y_*, *f_p_* are the resonant frequencies in drive, yaw, pitch/roll modes respectively; *f_obj_* = 14 kHz is the target frequency in the drive and sense modes.

Therefore, the mode matching process is converted to the optimization of the established objective function with minimum value by PSO algorithm. The resonant frequencies can be calculated by the springs stiffnesses and masses in the drive and sense modes:

(35) $$f_{d}=\sqrt{(2k_{1}+4k_{2}+4k_{3}+2k_{4}+4k_{5})/m_{d}}/2/\pi$$

(36) $$f_{y}=\sqrt{(2k_{6}+k_{7}+2k_{8}+4k_{9}+2k_{10})/m_{y}}/2/\pi$$

(37) $$f_{p}=\sqrt{(2k_{11}+2k_{12})/m_{p}}/2/\pi$$

where *k*_1_, *k*_2_, *k*_3_, *k*_5_ are the stiffnesses of relevant springs in drive axis; *k*_11_ and *k*_12_ are the stiffnesses of spring-11 and spring-12 in *z*-axis respectively.

The stiffnesses of different springs can be calculated as equations shown in Table 3 [4,24], where the Young’s modulus *E* = 1.3 × 10^11^ Pa; the shear modulus *G* = 7.96 × 10^10^ Pa; the coefficient *λ* = 0.089; the thickness of the out-of-plane decoupling spring *h_p_* = 30 μm.

According to Equation (33), the stiffness of spring-7 and -8 can be expressed by five unknown variables *k*_6_, *k*_9_, *k*_10_, *y_O_* and *y_E_* as:

(38) $$k_{7}+2k_{8}=\left[2k_{10}(y_{D}-y_{O})-2k_{9}(2y_{O}-y_{B}-y_{C})-2k_{6}(y_{O}-y_{E})\right]/(y_{O}-y_{A})$$

Based on the geometrical relationship of the gyroscope structure, the length of spring-5 can be expressed as:

(39) $$L_{5}=y_{E}-1.5w_{5}-70/\sqrt{2}-30$$

where the unit of all the variables are μm.

Similarly, the *y*-coordinate value of center of mass of the big frame can be calculated by dividing the frame into several simple geometric shapes. Thus, it is easy to get:

(40) $$y_{O}=\frac{\sum m_{i}y_{i}}{\sum m_{i}}=\frac{1.3339\times 10^{9}+136400y_{E}+9400w_{5}+L_{5}w_{5}y_{E}}{651440+L_{5}w_{5}}$$

where the unit of all the variables are μm.

To simplify the objective function, the variables can be assumed as:

(41) $$x(1)=\frac{w_{3}}{L_{3}},\;x(2)=\frac{w_{1}}{L_{1}},\;x(3)=\frac{w_{2}}{L_{2}},\;x(4)=\frac{w_{4}}{L_{4}},\;x(5)=\frac{w_{6}}{L_{6}},\;x(6)=\frac{w_{9}}{L_{9}},\;x(7)=\frac{w_{10}}{L_{10}},\;x(8)=\frac{{w_{11}}^{3}}{L_{11a}},\;x(9)=\frac{{w_{12}}^{3}}{L_{12a}},\;x(10)=w_{5},\;x(11)=y_{E}$$

**Table 3:** The stiffness expressions of different springs.

Springs	Dimensions	Stiffness Expressions
U-shaped springs: spring-1, -2, -4, -6, -9 and -10.	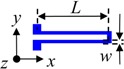	Stiffness in *y*-axis: $k_{y}=Eh{\left(w/L\right)}^{3}/2$
Double U-shaped spring: spring-3.	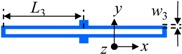	Stiffness in *y*-axis: $k_{3}=Eh{\left(w_{3}/L_{3}\right)}^{3}$
Double folded spring: spring-7.	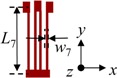	Stiffness in *x*-axis: $k_{7}=Eh{\left(w_{7}/L_{7}\right)}^{3}$
Combination of U-shaped spring and crab-leg spring: spring-5 and -6.	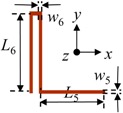	Stiffnesses in *x* and *y*-axes:$k_{6}=Eh{\left(w_{6}/L_{6}\right)}^{3}/2$, $k_{5}=\frac{Eh{\left(w_{5}/L_{5}\right)}^{3}(L_{5}-L_{6}/3.6)}{L_{5}+0.3L_{6}}$
Out-of-plane decoupling springs: spring-11 and -12.	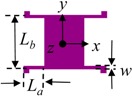	Stiffness in *z*-axis: $k_{z}=\frac{16\lambda Gh_{p}w^{3}}{L_{a}L_{b}^{2}}$

Thus stiffnesses of the springs can be expressed as:

(42) $$k_{3}=Ehx{\left(1\right)}^{3}/2,\;k_{1}=Ehx{\left(2\right)}^{3}/2,\;k_{2}=Ehx{\left(3\right)}^{3}/2,\;k_{4}=Ehx{\left(4\right)}^{3}/2\;k_{5}=Eh{\left(\frac{x(10)}{L_{5}}\right)}^{3}\frac{L_{5}-x(5)x(10)/3.6}{L_{5}+0.3x(5)x(10)},\;k_{6}=Ehx{\left(5\right)}^{3}/2,\;k_{9}=Ehx{\left(6\right)}^{3}/2\;k_{10}=Ehx{\left(7\right)}^{3}/2,\;k_{11}=Ehx{\left(8\right)}^{3}/2/L_{11b}^{2},\;k_{12}=Ehx{\left(9\right)}^{3}/2/L_{12b}^{2}$$

where *L*_11*b*_ and *L*_12*b*_ are 174 μm and 266 μm respectively. Substituting Equations (35–42) into Equation (34), the objective function can be expressed by variables *x*(*i*), where *i* = 1, 2, …, 11. The change ranges of these variables can be chosen by experience as:

(43) $$x(i)\in \left\{ \begin{array}{ll} \left[10/400,\;15/200\right] & i=1\\ \left[10/550,\;15/300\right] & i=2,3,\cdots 7\\ \left[6^{3}/180,\;10^{3}/60\right] & i=8,9\\ \left[20,\;80\right] & i=10\\ \left[2460,\;2660\right] & i=11 \end{array}\right.$$

The PSO algorithm can be divided into five steps as follows:

Step 1: Initialization of parameters. The changing ranges of the parameters are initialized as Equation (43); then a swarm of particles with *Size* = 100 are random initialized in the problem space. The maximum evolution generations *Gen* = 200. 

Step 2: According to the objective function, fitness of the particles *F*(*X_i_*) are evaluated, where *X_i_* denotes the location of a particle numbered *i* = 1, 2,…, *Size*. The previous best position *P_i_* of a particle itself and the global best position *BestS* of all the particles are reserved.

Step 3: Refresh the velocities and positions of the particles by:

(44) $$V_{i}^{kg+1}=bV_{i}^{kg}+c_{1}r_{1}(P_{i}^{kg}-X_{i}^{kg})+c_{2}r_{2}(BestS_{i}^{kg}-X_{i}^{kg})$$

(45) $$X_{i}^{kg+1}=X_{i}^{kg}+V_{i}^{kg+1}$$

where the evolution generation *kg* = 1, 2,…, *Gen*; particle number *i* = 1, 2,…, *Size*; the coefficients *c*_1_ = 1.5, *c*_2_ = 2.5 denote the strength of attraction towards the best positions; *r*_1_ and *r*_2_ are random numbers in the range of [0, 1].

Step 4: Refresh the best position *P_i_* of a particle itself and the global best position *BestS* of all the particles.

Step 5: Examine the end condition, and get back to Step 3 or terminate the process.

**Figure 7:**
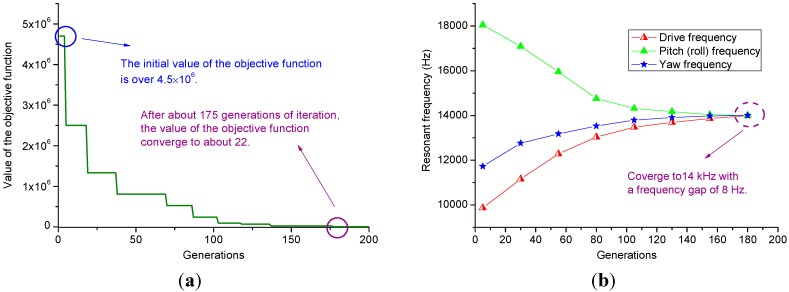
Results of the PSO algorithm. (**a**) Value of the objective function *versus* generations; (**b**) Resonant frequencies obtained by calculation *versus* generations.

Based on the discussions above, the PSO algorithm can be easily implemented in Matlab. The obtained results are shown in Figure 7 and Table 4. In Figure 7b, the resonant frequencies in drive and sense modes are calculated by Equations (35–37). 

**Table 4:** The optimized values of the variables in PSO algorithm.

*x*(1)	*x*(2)	*x*(3)	*x*(4)	*x*(5)	*x*(6)	*x*(7)	*x*(8)	*x*(9)	*x*(10)	*x*(11)
0.025	0.0405	0.0395	0.0405	0.0415	0.0357	0.0385	3.531	3.531	60	2502

Based on the optimized data and our experiences on the design of gyroscopes, the springs dimensions can be chosen as:

(46) $$\frac{w_{3}}{L_{3}}=\frac{10}{400},\;\frac{w_{1}}{L_{1}}=\frac{15}{370},\;\frac{w_{2}}{L_{2}}=\frac{15}{380},\;\frac{w_{4}}{L_{4}}=\frac{15}{370},\;\frac{w_{6}}{L_{6}}=\frac{60}{1445},\;\frac{w_{9}}{L_{9}}=\frac{15}{420}\;\frac{w_{10}}{L_{10}}=\frac{15}{390},\;\frac{{w_{11}}^{3}}{L_{11a}}=\frac{8^{3}}{145},\;\frac{{w_{12}}^{3}}{L_{12a}}=\frac{8^{3}}{145},\;w_{5}=60,\;y_{E}=2502$$

Substituting Equation (46) into Equations (38–40), dimensions of spring-7 and spring-8 can be chosen as:

(47) $$\frac{w_{7}}{L_{7}}=\frac{10}{430},\;\frac{w_{8}}{L_{8}}=\frac{13}{380}$$

To verify the accuracy of PSO algorithm, the obtained springs dimensions are substituted into the established model in ANSYS. The modal simulation results show that the resonant frequencies are 13,967 Hz, 14,035 Hz and 14,116 Hz in drive, yaw and pitch/roll modes respectively. Obviously, there is a frequency split of 149 Hz, which is larger than the calculation results shown in Figure 7b. This may be explained by the fact that the stiffness calculation equations of the springs used in the PSO algorithm cannot ideally reflect the real situation.

### 3.2. Further Mode Matching by Experiences

To further match the drive and sense modes frequencies, the relevant spring dimensions can be iteratively adjusted by calculation and experience. Because the resonant frequencies are already near the target frequency of 14 kHz, the lengths of the springs are slightly adjusted to achieve mode matching. 

**Figure 8:**
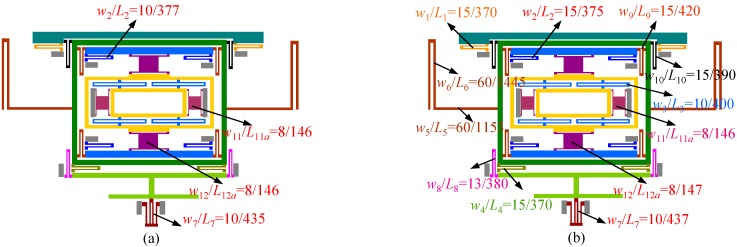
Further mode matching process can be divided into two steps (the changed dimensions are marked in red): (**a**) step 1: length of spring-2, spring-7, spring-11 and spring-12 are adjusted; (**b**) step 2: length of spring-2, spring-7 and spring-12 are further changed.

**Figure 9:**
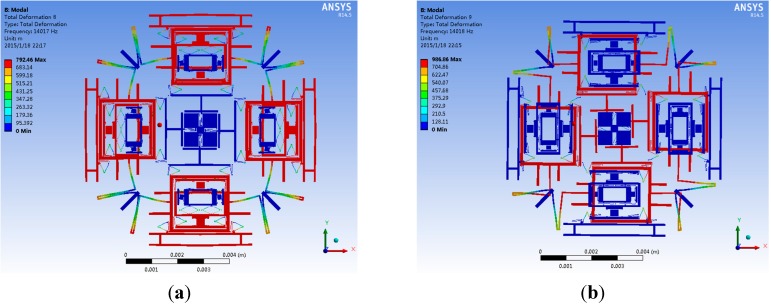

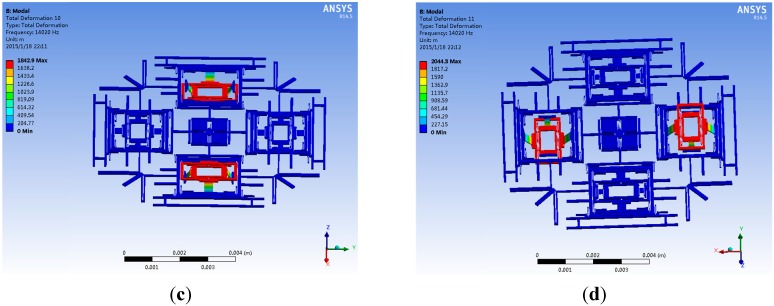
The modal analysis results of the tri-axis gyroscope: (**a**) The drive mode; (**b**) The yaw mode; (**c**) The pitch mode; (**d**) The roll mode.

This manual frequency adjusting process can be divided into two steps as shown in Figure 8a,b. After the first step change of the springs dimensions, resonant frequencies in drive, yaw and pitch/roll modes are 13,984 Hz, 14,025 Hz and 14,057 Hz respectively. Then by elaborately changing the springs dimensions in the second step, the natural frequencies in drive, yaw and pitch/roll modes can be matched to 14,017 Hz, 14,018 Hz and 14,020 Hz, respectively. The modal analysis results are shown in Figure 9. Obviously, a frequency gap of 3 Hz is achieved in theory.

## 4. Simulation Results

### 4.1. Cross-Axis Effect Analysis

To improve the performance of a tri-axis gyroscope, it is essential to avoid the coupling between the drive and sense modes. Thus, the structure is designed to be fully decoupled to minimize the coupling effect at the most extent. However, cross-axis effect still exists due to the unavoidable deformation of the decoupling springs. The amplitude of movement in drive mode can be up to 10 μm, which is several orders of magnitude larger than that of the sense modes. Thus, the main cross-axis effect appears to be the drive-to-sense coupling. 

**Figure 10:**
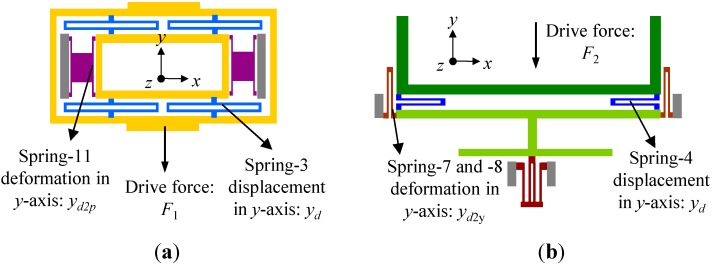
Simplified models of drive-to-sense coupling effect: (**a**) Drive-to-pitch coupling effect; (**b**) Drive-to-yaw coupling effect.

The drive-to-pitch/roll coupling effect can be simplified as the model shown in Figure 10a. In an ideal situation, the inner frame in pitch/roll mode has only 1-DOF in *z*-axis. However, the stiffness of spring-11 in *y*-axis is not infinite, which means that it is inevitable to deform in the drive direction by the force transformed from spring-3. Thus the sense frame will show an unwanted movement in *y*-axis, resulting in the coupling of drive mode to pitch/roll mode. Assuming that the outer frame in pitch/roll mode is driven to oscillate with an amplitude of *y_d_* in *y*-axis, the drive-to-pitch coupling displacement can be simply expressed as:

(48) $$y_{d2p}=\frac{F_{1}}{2k_{11y}}=\frac{4k_{3}y_{d}}{2k_{11y}}=\frac{2k_{3}y_{d}}{k_{11y}}$$

where *k*_11*y*_ is the stiffness of spring-11 in *y*-axis; *F*_1_ = 4*k*_3_*y_d_* is the drive force.

Since the springs dimensions have been chosen in Section 3, the stiffnesses of them in all the three axes can be easily calculated. Therefore, the drive-to-pitch coupling ratio can be deduced from Equation (48) as:

(49) $$\frac{y_{d2p}}{y_{d}}=\frac{2k_{3}}{k_{11y}}=0.71\%$$

Similarly, the simplified model of drive-to-yaw coupling effect is shown in Figure 10b. The drive-to-yaw coupling displacement can be expressed as:

(50) $$y_{d2y}=\frac{F_{2}}{k_{7y}+2k_{8y}}=\frac{2k_{4}y_{d}}{k_{7y}+2k_{8y}}$$

where *k*_7*y*_ and *k*_8*y*_ are the stiffnesses of spring-7 and spring-8 in *y*-axis respectively; *F*_2_ = 2*k*_4_*y_d_* is the drive force applied on the outer frame in pitch/roll mode.

The drive-to-yaw coupling ratio can be deduced from Equation (50) as:

(51) $$\frac{y_{d2y}}{y_{d}}=\frac{2k_{4}}{k_{7y}+2k_{8y}}=0.13\%$$

The coupling effect can be simulated using ANSYS static structural analysis. By applying a displacement load on the drive frames in drive axis, the displacement of the sense frames in drive axis can be found by the simulation software. The simulation results of the cross-axis effect are listed in Table 5. 

Like the drive-to-sense coupling simulation process, the sense mode coupling is proved to be nearly zero. Thus the coupling effect between the sense modes can be ignored in the analysis. Moreover, the overlapping areas of the sensing combs/plates in yaw and pitch/roll modes are not affected by the displacement in drive direction. Therefore, the sensing capacitances are free of the drive-to-sense coupling.

**Table 5:** Simulation results of cross-axis effect.

Different Sense Modes	Drive Amplitude (m)	Coupling Displacement (m)	Drive-to-Sense Coupling Ratio
Pitch/roll	1 × 10^−5^	7.18 × 10^−8^	0.71%
Yaw	1 × 10^−5^	1.42 × 10^−8^	0.13%

### 4.2. Quadrature Error Correction and Frequency Tuning Analysis

**Figure 11:**
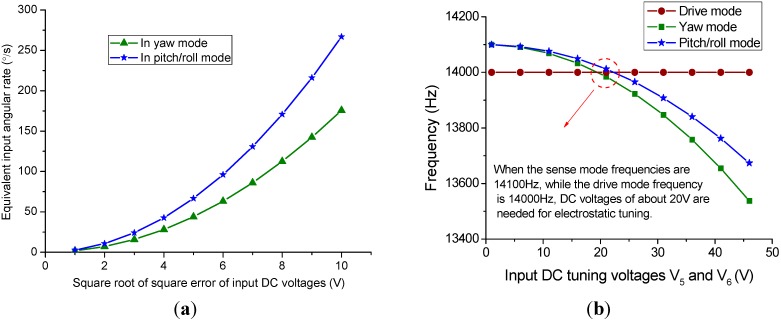
Simulation results of (**a**) quadrature error correction and (**b**) frequency tuning.

According to Equations (5) and (8), the quadrature error in the yaw and pitch/roll modes can be corrected by applying appropriate DC voltages on the quadrature electrodes. To simplify the analysis, the measured quadrature error signal can be treated as an input angular rate [25]. Based on the parameters listed in Table 2, the simulation result of the quadrature error correction effect can be depicted in Figure 11a. The horizontal ordinate is the square root of input voltages *V*_1_^2^ − *V*_2_^2^ or *V*_3_^2^ − *V*_4_^2^, which is assumed to be in the range of [1,10] V. The vertical ordinate represents the equivalent input angular rate of quadrature error that the input DC voltages can correct. It is shown from the simulation result that the equivalent input angular rates that can be corrected in yaw and pitch/roll modes with *V*_2_ = *V*_4_ = 0 V, *V*_1_ = *V*_3_ = 10 V are 175 °/s and 266 °/s respectively.

Like the quadrature error correction method, the frequency tuning can be achieved by applying the needed DC voltages on the stiffness tuning electrodes. According to the negative electrostatic stiffness tuning expressions shown in Equations (11) and (13), it is easy to find the relationship of tuning frequency and input DC voltage as shown in Figure 11b. With an applied voltage of *V*_5_ = 19.5 V and *V*_6_ = 22.3 V, the frequencies in yaw and pitch/roll modes are well matched to drive mode of 14,000 Hz from the assumed start frequency splits of 100 Hz.

### 4.3. Sensitivity Analysis

**Figure 12:**
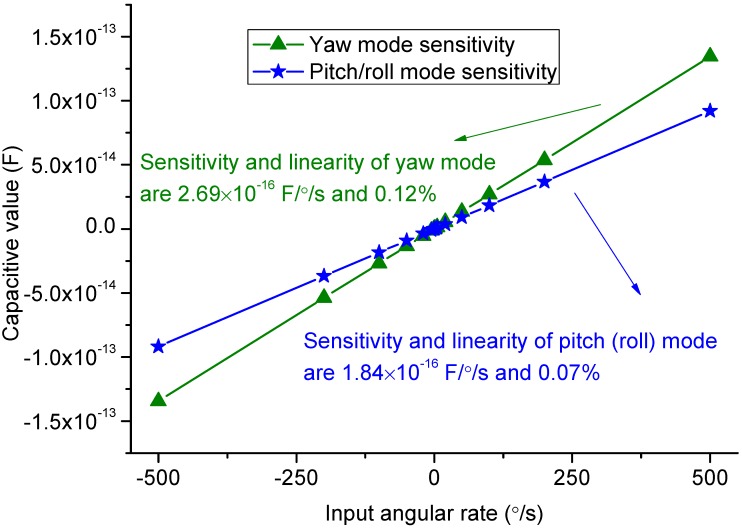
Sensitivity simulation results.

The scale factor of the gyroscope can be simulated by harmonic response analysis in ANSYS. With the input angular rates varied from −500 to 500 °/s, the displacements of the sense frames can be calculated from simulation. Converting the obtained displacements into capacitive values, the scale factor of the sense modes can be depicted in Figure 12. 

The linearities of the yaw mode and pitch/roll mode are 0.12% and 0.07%, respectively. Some assumed parameters for simulation are listed in Table 6. Because the yaw mode is detected by parallel plates and the pitch/roll mode is comb sensing, the quality factor in yaw mode is assumed to be less than that in pitch mode. The simulation results are summarized in Table 7. The mechanical Brownian noise floor of the sense modes are calculated at the temperature of 20 °C [21].

**Table 6:** Some assumed parameters for simulation.

Assumed Q-Factors in Different Modes			Assumed Drive Voltages (V)	
Drive (*Q_d_*)	Yaw (*Q_y_*)	Pitch/roll (*Q_p_*)	DC voltage	AC voltage
2000	500	1000	5	5

**Table 7:** Simulation results.

Simulation Results	Drive Mode	Yaw Mode	Pitch/Roll Mode
Natural frequency	14,017 Hz	14,018 Hz	14,020 Hz
Amplitude	6.44 × 10^−6^ m	4.07 × 10^−10^ m/°/s	7.62 × 10^−10^ m/°/s
Capacity sensitivity	3.40 × 10^−8^ F/m	2.69 × 10^−16^ F/°/s	1.84 × 10^−16^ F/°/s
Sense linearity	/	0.12%	0.07%
Brownian noise floor	/	0.18 °/hr/√Hz	0.17 °/hr/√Hz

### 4.4. Bias Drift Estimation

The sources of bias drift can be divided into two parts. One is the sensor element, the other is the interface electronics [26]. To estimate the bias drift of the proposed tri-axis gyroscope in this paper, the source of sensor element is analyzed. It is known that the quadrature error is mainly responsible for the bias drift of a gyroscope. When the quadrature error is taken into consideration, the kinetic equations of the tri-axis gyroscope can be expressed as:

(52) $$\ddot{x}+\frac{Q_{d}}{m_{d}}\dot{x}+\omega_{d}^{2}x=\frac{F_{d}}{m_{d}}\sin(\omega_{d}t)$$

(53) $$\ddot{y}+\frac{Q_{y}}{m_{y}}\dot{y}+\omega_{y}^{2}y=\frac{F_{qy}}{m_{y}}-2\Omega_{y}\dot{x}$$

(54) $$\ddot{p}+\frac{Q_{p}}{m_{p}}\dot{p}+\omega_{p}^{2}p=\frac{F_{qp}}{m_{p}}-2\Omega_{p}\dot{x}$$

where *x*, *y*, *p* are displacements in the drive, yaw, pitch/roll modes respectively; *ω_d_*, *ω_y_*, *ω_p_* are the angular frequencies in the drive, yaw, pitch/roll modes respectively; *F_d_* is the drive force. Ω*_y_*, Ω*_p_* are the input angular rates in the yaw, pitch/roll modes respectively; *F_qy_*, *F_qp_* are the quadrature forces in the yaw, pitch/roll modes, respectively.

According to Equations (52)~(54), the expressions of *x*, *y*, *p* can be solved as:

(55) $$x=\frac{F_{d}Q_{d}}{\omega_{d}^{2}}\sin(\omega_{d}t-\frac{\pi }{2})$$

(56) $$y=\underset{From\;Coriolis\;force}{\underset{︸}{\frac{2m_{y}\Omega_{y}F_{d}Q_{d}Q_{y}}{\omega_{d}^{2}\omega_{y}}\cos(\omega_{d}t+\varphi )}}+\underset{From\;quadrature\;error}{\underset{︸}{\frac{k_{yx}F_{d}Q_{d}Q_{y}}{\omega_{d}^{2}\sqrt{{\left(\omega_{y}^{2}-\omega_{d}^{2}\right)}^{2}+\omega_{y}^{2}\omega_{d}^{2}/Q_{y}^{2}}}\sin(\omega_{d}t+\varphi )}}$$

(57) $$p=\underset{From\;Coriolis\;force}{\underset{︸}{\frac{2m_{p}\Omega_{p}F_{d}Q_{d}Q_{p}}{\omega_{d}^{2}\omega_{p}}\cos(\omega_{d}t+\theta )}}+\underset{From\;quadrature\;error}{\underset{︸}{\frac{k_{px}F_{d}Q_{d}Q_{p}}{\omega_{d}^{2}\sqrt{{\left(\omega_{p}^{2}-\omega_{d}^{2}\right)}^{2}+\omega_{p}^{2}\omega_{d}^{2}/Q_{p}^{2}}}\sin(\omega_{d}t+\theta )}}$$

(58) $$\varphi =\arctan\frac{(\omega_{y}^{2}-\omega_{d}^{2})Q_{y}}{\omega_{y}\omega_{d}},\theta =\arctan\frac{(\omega_{p}^{2}-\omega_{d}^{2})Q_{p}}{\omega_{p}\omega_{d}}$$

where *k*_y*x*_, *k_px_* are the coupling stiffnesses from the drive mode to the yaw and pitch/roll modes respectively.

**Figure 13:**
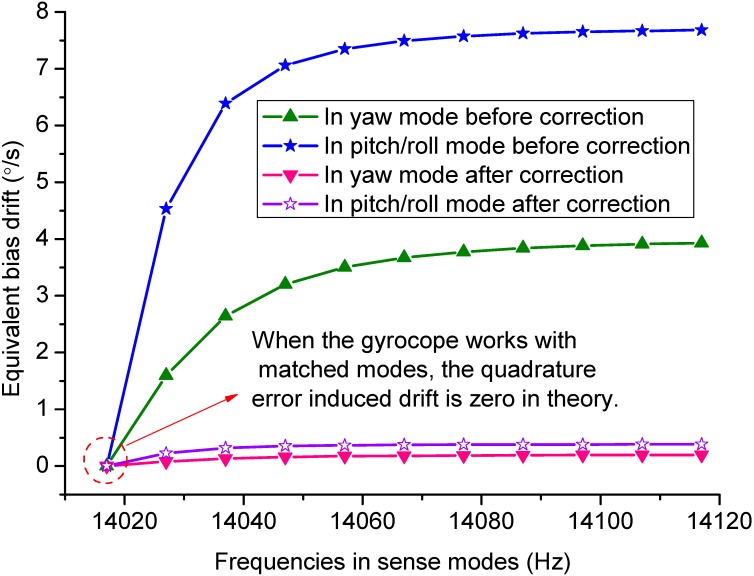
Equivalent bias drift *versus* sense mode frequencies before and after quadrature error correction.

Assuming that the coupling stiffnesses *k*_y*x*_ = *k_px_* = 1 N/m, the bias drift can be calculated by the multiplication demodulation method according to the displacement expressions in Equations (55)~(58). The bias drift can be described as equivalent input angular rates around the sense directions. The relationship of bias drift and the frequency split in the drive and sense modes are depicted in Figure 13. It is assumed that the quadrature errors are reduced to 5% of the original values after correction. Thus the equivalent bias drifts are 7.6 °/s and 3.9 °/s before quadrature error correction, 0.4 °/s and 0.2 °/s after quadrature error correction in the yaw and pitch/roll modes, respectively.

## 5. Conclusions and Future Prospect

Based on the theoretical analysis and previous studies on the multi-axis gyroscope, a novel tri-axis gyroscope with fully decoupled structure design is proposed and analyzed. The decoupling of drive and sense modes are achieved by elaborately arranging various springs, including the U-shaped spring, double U-shaped spring, double folded spring and out-of-plane decoupling spring. To improve the performance of the tri-axis gyroscope, the quadrature error correction electrodes, stiffness tuning electrodes and feedback electrodes are taken into consideration in all the sense modes in the structure design. The dimensions of the frames/beams in the drive and sense modes are obtained by carefully calculating the drive force, sensitivities and masses in different modes. The mechanism of the unwanted rotational mode is described and analyzed, which provides a direction for choosing the springs dimensions. To speed up the mode matching process, the PSO algorithm is adopted to choose the sizes of the springs. By this algorithm, the frequency split of the drive and sense modes can be matched with a gap of 149 Hz. The frequency split is further decreased to 3 Hz by manual adjusting the relevant spring dimensions. The ANSYS simulation results show that the coupling rations of the drive and sense modes are all less than 0.2%. Besides, the scale factors in the yaw and pitch/roll modes are 0.269 fF/°/s and 0.184 fF/°/s when assuming that their quality factors are 500 and 1000, respectively. 

Future efforts will be put into the fabrication and test of the proposed tri-axis gyroscope. These plans will been arranged in the next phase though it will take a long time. In fact, there are some challenging facts in this process. First, it is not easy to precisely and simultaneously control the 15 μm depth of positive and negative trenches for out-of-plane comb fingers and decoupling springs. This parameter is significantly related to the stiffness of pitch/roll mode and the equivalent mass. Second, the expected sub 1 μm dual face alignment precision between trench and comb fingers is of crucial importance to the success of process. These two main problems oblige us to utilize high precision equipment with the aid of optimal experienced technicians. Third, due to the complexity of the electrode numbers, more than 60 for many functions, compared with other designs, the complete wire bonding must be given careful consideration for PCB layout design to avoid undesired parasitic capacitances. Though the above problems can be totally overcome by many attempts, the fabrication errors are still unavoidable and will result in many performance degradations. Naturally, the verification by test is not reliable and convincing here. We think an ideal option would be to adopt CMOS-MEMS combination technology, regardless of the fact that it is out of our research scope. Obviously, it will be the main trend for eventual batch production for industrial applications.
